# PCBP2 post‐transcriptionally regulates sortilin expression by binding to a C‐rich element in its 3′ UTR

**DOI:** 10.1002/2211-5463.12794

**Published:** 2020-02-03

**Authors:** Toshiki Yabe‐Wada, Caroline C. Philpott, Nobuyuki Onai

**Affiliations:** ^1^ Department of Immunology Kanazawa Medical University Kahoku Uchinada Japan; ^2^ Liver Diseases Branch National Institute of Diabetes and Digestive and Kidney Diseases National Institutes of Health Bethesda MD USA

**Keywords:** cytokines, innate immunity, post‐transcriptional regulation, RNA‐binding proteins, *Sort1*, zinc

## Abstract

Post‐transcriptional regulation of cytokine production is crucial to ensure appropriate immune responses. We previously demonstrated that poly‐rC‐binding protein‐1 (PCBP1) can act as a *trans*‐acting factor to stabilize transcripts encoding sortilin, which mediates cytokine trafficking. Here, we report that PCBP2, which strongly resembles PCBP1, can stabilize sortilin transcripts in macrophages using the same mechanism employed by PCBP1. PCBP2 recognized the C‐rich element in the 3′ UTR of sortilin mRNA, and PCBP2 knockdown decreased sortilin transcripts, indicating that PCBP2 stabilizes sortilin mRNA by binding to its 3′ UTR. Zn^2+^ reversibly inhibited the nucleotide binding ability of PCBP2 *in vitro*. These findings suggest that both PCBP2 and PCBP1 may control the stability of sortilin transcripts by sensing intracellular Zn^2+^ levels in immune cells.

## Graphical abstract

We analyzed the function of poly‐rC‐binding protein‐2 (PCBP2), a paralog of PCBP1, in stabilizing the mRNA of sortilin, a cytokine trafficking mediator. PCBP2 binds to C‐rich elements in the 3′ UTR of sortilin mRNA; Zn^2+^ interferes with this interaction, suggesting that both PCBP1 and PCBP2 may regulate the stability of sortilin mRNA by sensing the intracellular Zn^2+^ levels.

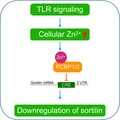

**Abbreviations**
cDC: conventional dendritic cell
CRE: C‐rich element
PCBP: Poly‐rC‐binding protein
pDC: plasmacytoid dendritic cell
PRR: pattern‐recognition receptor
RBP: RNA‐binding protein
TLR: Toll‐like receptor

Recognition of pathogen‐associated molecular patterns by pattern‐recognition receptors (PRRs) and the subsequent production of proinflammatory cytokines play an important role in the innate immune system 1. Excessive immune responses are characterized by the overproduction of cytokines, which can cause tissue damage by chronic inflammation and lead to autoimmune disease. Therefore, tight control of the signaling pathways regulating cytokine production is important for appropriate immune responses.

RNA‐binding proteins (RBPs) contribute to the post‐transcriptional regulation of immunity‐related mRNA 2; this regulation is important for the repression of excessive immune responses 3. Specific *cis*‐elements for post‐transcriptional regulation identified in the 3′UTRs of many immunity‐related mRNA contribute to their degradation or stabilization; moreover, many different RBPs, as *trans*‐acting factors involved in this regulation, have been identified 2, 4. The ARE‐binding proteins tristetraprolin, AUF1, and HuR recognize and bind to mRNA containing AU‐rich elements, which results in the control of target mRNA stability. Roquin and Regnase‐1 bind to stem‐loop structures followed by destabilization of target mRNA 2, 3, 4. The functions of these RBPs are regulated by a number of kinase pathways, resulting in the coordinated expression of their target mRNA 2, 3.

Poly‐rC‐binding proteins (PCBPs) are a group of multifunctional RBPs. They recognize C‐rich elements (CREs) of single‐stranded RNA and have diverse functions affecting RNA processing, translation, and stability through their binding to CREs 5, 6. A recent study demonstrated that *Pcbp1*‐deficient mouse embryos die at the peri‐implantation stage 7, indicating an essential role of PCBP1 in mouse embryonic development. PCBPs are also important for iron metabolism, acting as iron chaperones 8, 9, 10, 11, 12, 13, 14. Moreover, PCBP1 mediates proinflammatory cytokine production via stabilization of mRNA in iron‐promoted CD4^+^ T‐cell pathogenicity 15. In microRNA processing, PCBP2 functions by sensing cytosolic iron status 16. These studies suggest that PCBP1 and PCBP2 link RNA stability and processing to iron metabolism.

We previously demonstrated that PCBP1 is involved in the regulation of sortilin, which mediates cytokine trafficking via stabilization of its mRNA 17. Interestingly, the binding of Zn^2+^ to PCBP1 reversibly interfered with the nucleotide binding activity of PCBP1 *in vitro* 17. Two major isoforms, PCBP1 and PCBP2, are ubiquitously expressed 18. They are highly homologous; however, little is known about the involvement of PCBP2 in the post‐transcriptional regulation of sortilin or whether Zn^2+^ affects the nucleotide binding activity of PCBP1 paralogs. In this study, we measured the protein expression levels of PCBP1 and PCBP2 in multiple types of immune cells and examined the involvement of PCBP2 in the stabilization of sortilin mRNA. We describe the interaction of PCBP2 with the CRE of the 3′ UTR of sortilin mRNA, which leads to the stabilization of the sortilin mRNA. Zn^2+^ reversibly interferes with PCBP2 nucleotide binding to the CRE. We previously observed that loading primary macrophages with Zn^2+^ leads to a reduction of sortilin mRNA 17, suggesting that PCBP2, like PCBP1, may control sortilin mRNA stability by sensing intracellular Zn^2+^ levels in immune cells. Our observations provide insights into the roles of PCBP1 and PCBP2 in mRNA regulation, metal homeostasis, and innate immunity via the post‐transcriptional control of sortilin expression.

## Results and Discussion

### PCBP1 and PCBP2 are expressed in multiple types of immune cells

**Figure 1:**
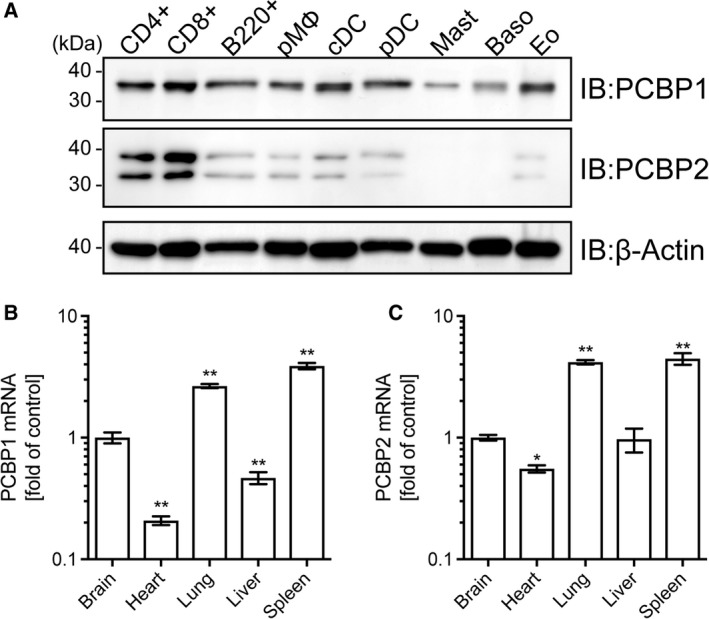
Expression profiles of PCBP1 and PCBP2 in immune cells and tissues. (A) Expression of PCBP1 and PCBP2 protein. β‐Actin was used as an internal control. CD4^+^, splenic CD4^+^ cells; CD8^+^, splenic CD8^+^ cells; B220^+^, splenic B220^+^ cells; Baso, bone marrow‐derived basophils; Eo, bone marrow‐derived eosinophils; Mast, bone marrow‐derived mast cells; pMØ, peritoneal macrophages. IB denotes the antibody used for immunoblotting. (B, C) Expression of *Pcbp1* (B) and *Pcbp2* (C) mRNA in mouse organs quantified by qRT‐PCR, normalized to *Gapdh* expression. Data are the means ± SD (*n* = 3). *P < 0.05 **P < 0.01 (two‐way ANOVA).

To investigate the expression levels of PCBP1 and PCBP2 proteins in murine immune cells, we carried out immunoblot analysis, indicating that PCBP1 and PCBP2 were expressed at high levels in CD4^+^ and CD8^+^ splenocytes (Fig. 1A). These proteins were moderately expressed in B220^+^ splenocytes; peritoneal macrophages; and bone marrow‐derived conventional dendritic cells (cDCs), plasmacytoid DCs (pDCs), and eosinophils (Fig. 1A). PCBP1 was modestly expressed in bone marrow‐derived mast cells and basophils; in contrast, there was no visible PCBP2 expression in mast cells or basophils. We detected endogenous PCBP2 as a doublet band. A doublet band has been reported using other antibodies, and immunoblot analysis with mouse PCBP2 knockout embryos has shown complete loss of the doublet band 7. This doublet band was depleted in PCBP2 knockdown cells with siRNA (Fig. 3A), indicating that the two detected bands were derived from endogenous PCBP2 rather than being nonspecific bands. Various *Pcbp2* transcript variants registered in the database supports this view. Interestingly, we previously found lowered expression levels of sortilin in mast cells 17, correlating with these PCBP1 and PCBP2 expression levels, suggesting that these proteins are not important for cytokine secretion from mast cells. Quantitative real‐time PCR (qRT‐PCR) with tissue samples showed that *Pcbp1* mRNA was expressed at high levels in the brain, lungs, and spleen, with lower levels in the heart and liver (Fig. 1B). *Pcbp2* mRNA was predominantly expressed in the lung and spleen, while very similar *Pcbp2* mRNA expression levels were observed in the brain, heart, and liver (Fig. 1C). Overall, the expression patterns of *Pcbp1* and *Pcbp2* are similar to that of sortilin as we previously reported 17.

### PCBP2 stabilizes sortilin mRNA

**Figure 2:**
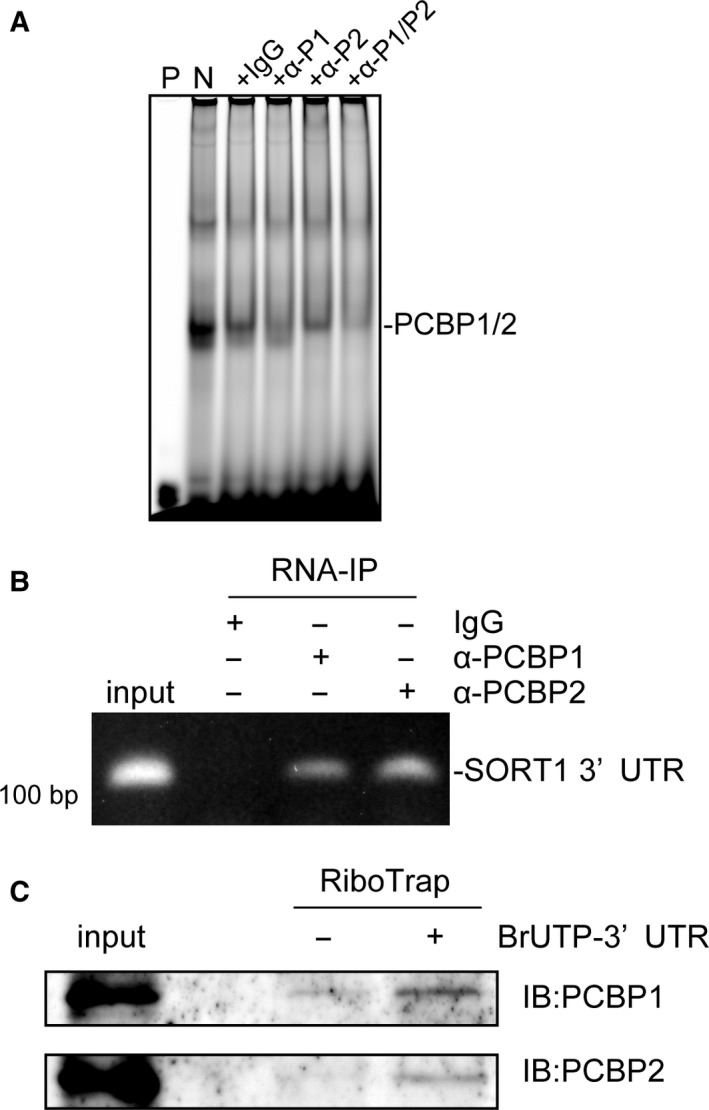
PCBP2 binds to CREs in the 3′ UTR of sortilin mRNA. (A) RNA EMSA analysis. P, CRE probe mixed with binding buffer alone; N, whole‐cell macrophage lysates mixed with labeled WT probe and 50‐fold molar excess of nonspecific competitor; +IgG, negative control; +α‐P1, anti‐PCBP1 antibody supershift; +α‐P2, anti‐PCBP2 supershift; +α‐P1/P2, anti‐PCBP1 and anti‐PCBP2 supershift. (B) Sortilin mRNA coimmunoprecipitates with both PCBP1 and PCBP2. (C) RiboTrap analysis. Macrophage cytosol was mixed with the BrUTP‐labeled 3′ UTR synthesized *in vitro*. Protein/RNA complexes were precipitated with protein A/G magnetic beads bound to anti‐BrdU or rabbit IgG at 4 °C for 3 h. The BrUTP‐labeled RNA–protein complex was eluted with BrdU in PBS, followed by immunoblotting.

We next investigated the involvement of PCBP2 in the post‐transcriptional regulation of sortilin expression. We carried out an RNA electromobility shift assaywith macrophage lysates and an RNA probe containing the sortilin CREs as previously described 17. Major doublet band was found in the negative control sample (lane N in Fig. 2A). Interestingly, the observed upper or lower band was mostly supershifted following incubation with anti‐PCBP1 or anti‐PCBP2, respectively, and co‐incubation with both antibodies supershifted these doublet bands. Meanwhile, the control incubated with normal IgG did not show supershifted bands (Fig. 2A). These observations indicate that the upper or lower protein–RNA complex mainly contains PCBP1 or PCBP2, respectively, suggesting both PCBP1 and PCBP1 can independently bind CREs. We observed, by RNA immunoprecipitation (RIP), that CRE‐containing fragments in the 3′ UTR of sortilin mRNA were amplified in the samples immunoprecipitated with anti‐PCBP2, as were those immunoprecipitated with anti‐PCBP1 (Fig. 2B), indicating that endogenous PCBP2 directly interacts with the sortilin CRE. Moreover, immunoprecipitation with macrophage cell lysate coprecipitated endogenous PCBP2 in addition to endogenous PCBP1 with BrUTP‐labeled RNA derived from the 3′ UTR of the sortilin mRNA (Fig. 2C), indicating the capability of both PCBP1 and PCBP2 to bind. These data strongly suggest that PCBP2 directly interacts with the CRE in the 3′ UTR of sortilin mRNA.

**Figure 3:**
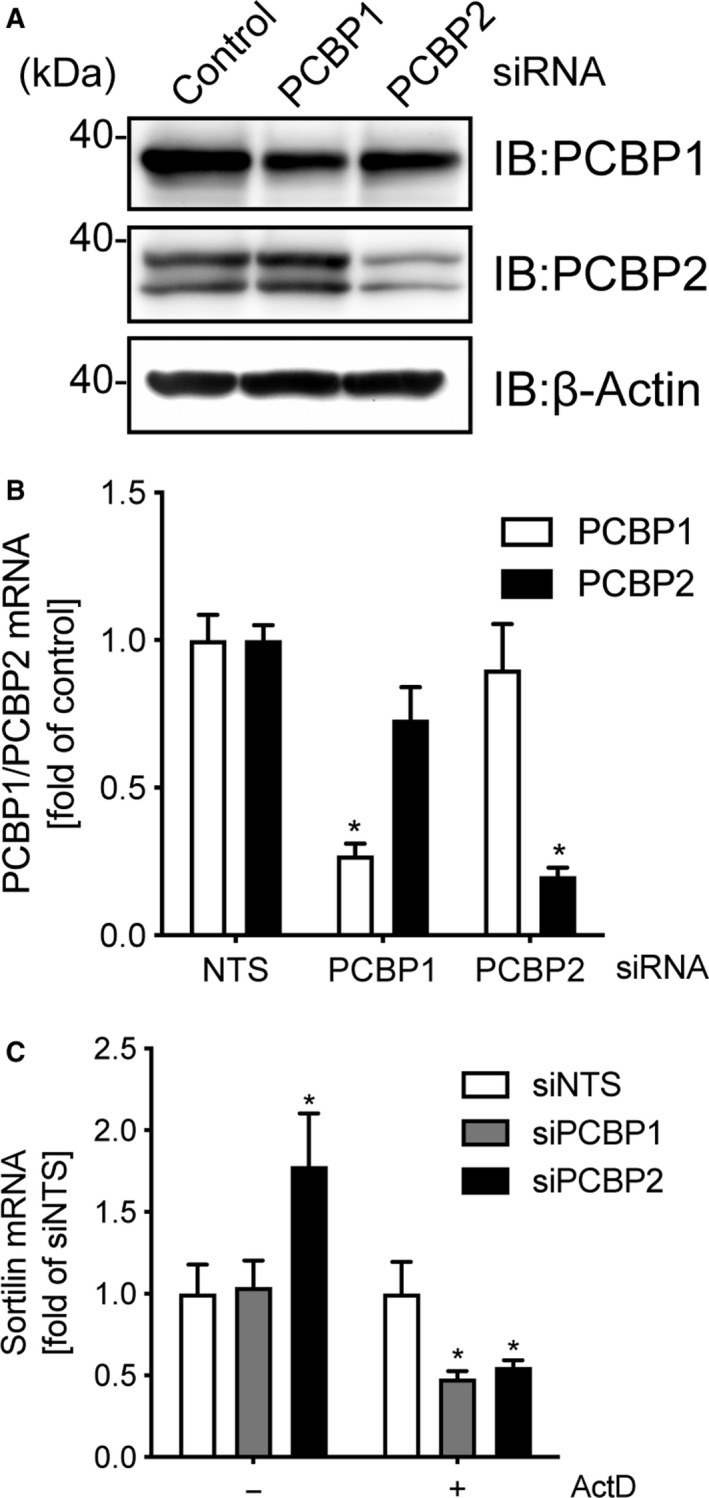
PCBP2 depletion affects sortilin mRNA levels. (A, B) Depletion of PCBP1 and PCBP2 in RAW264.7 cells. Cells were transfected with siRNA targeting *Pcbp1*, *Pcbp2*, or a control nontargeting siRNA, then harvested 48 h after transfection. Cell lysates were subjected to immunoblotting (A) with the indicated antibodies. Total RNA were subjected to qRT‐PCR (B) to quantify *Pcbp1* and *Pcbp2* mRNA. Data are the means ± SD (*n* = 3). *P < 0.01 (two‐way ANOVA). (C) Destabilization of sortilin mRNA by PCBP1 depletion in cells. RAW264.7 cells were transfected with control (siNTS), PCBP1 (siP1), or PCBP2 (siP2) siRNA for 48 h and, then, treated with 1 µg·mL^−1^ ActD for 9 h. Total RNA were subjected to qRT‐PCR to quantify sortilin mRNA. Data are the means ± SD (*n* = 3). *P < 0.01 (two‐way ANOVA).

To address the question whether PCBP2 stabilizes sortilin mRNA, we depleted PCBP2 in the murine macrophage cell line RAW264.7 using siRNA, followed by quantitation of sortilin mRNA by qRT‐PCR. We confirmed that siRNA‐transfected cells exhibited reduced PCBP1 and PCBP2 expression at both the mRNA and protein levels (Fig. 3A,B). We observed that both PCBP1 and PCBP2 knockdown with actinomycin D (ActD) treatment efficiently decreased sortilin mRNA levels compared with non‐targeting siNTS; however, this difference was attenuated without ActD treatment (Fig. 3C), consistent with the destabilization of sortilin mRNA by PCBP2 depletion. Collectively, these data suggest that PCBP2 promotes the stabilization of sortilin transcripts by binding to the CRE in the 3′ UTR of sortilin mRNA.

### Zn^2+^ affects the nucleotide binding activity of PCBP2

**Figure 4:**
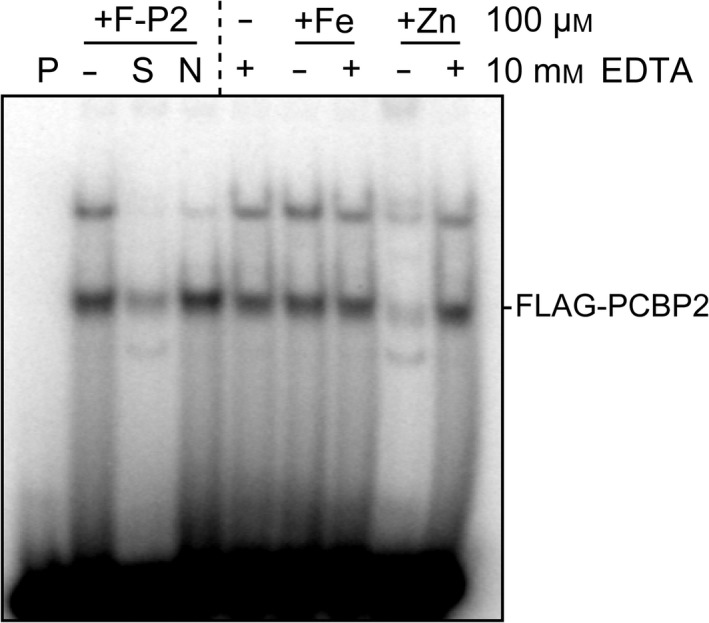
Zinc ions affect the poly(C)‐binding ability of PCBP2. EMSA analysis of PCBP2. ^32^P‐labeled poly(C) oligonucleotide probe (100 nm) was mixed with buffer alone (P) or yeast lysates expressing FLAG‐PCBP2. Samples were separated by PAGE. A 10‐fold molar excess of unlabeled poly(C) oligonucleotide or control mutated oligonucleotide was added as specific competitor (S) and nonspecific competitor (N), respectively. Zinc sulfate and ferrous ammonium sulfate (both 100 μm) with or without 10 mm EDTA were incubated with samples prior to incubation with the probes.

We previously reported reversible inhibition of nucleotide binding ability of PCBP1 by Zn^2+^ 17. To investigate whether the poly(C)‐binding activity of PCBP2 is also affected by Zn^2+^, we performed electrophoretic mobility shift assay (EMSA) on yeast cell lysates expressing FLAG‐PCBP2 in the presence of Zn^2+^. As expected, the shifted band derived from the complex of FLAG‐PCBP2 with radiolabeled probe was decreased when the lysate was incubated with zinc, whereas the signals were unchanged when the lysate was incubated with iron, indicating the Zn^2+^ specificity of PCBP2 binding (Fig. 4). Incubation with an excess amount of EDTA attenuated this binding (Fig. 4), indicating a reversible inhibition of PCBP2 binding. This reversible inhibition is consistent with the requirement of Zn^2+^ for PCBP2 poly(C) binding.

Pathogen infection and cellular stimuli induce PRR signaling, resulting in cytokine and chemokine production by immune cells. These inflammatory responses protect the body against harmful stimuli; however, chronic inflammation and/or autoimmune diseases are primarily attributed to excessive immune responses caused by cytokine overproduction. Therefore, the appropriate regulation of cytokine production is crucial for accurate immune responses. Here, we demonstrate that PCBP2, a paralog of PCBP1, can control the stability of sortilin mRNA through the binding to the CRE in 3′ UTR, along with PCBP1. So far, it has been reported that sortilin plays a key role in exocytic trafficking of cytokines in multiple types of immune cells 17, 19, 20, suggesting the involvement of both PCBP2 and PCBP1 in the post‐translational regulation of cytokine production through sortilin.

Toll‐like receptor (TLR) stimulation leads to a decrease in the amount of sortilin mRNA that is stabilized by PCBP1 17 and PCBP2 (Fig. 3), suggesting that both PCBPs have important roles in the regulation of sortilin mRNA by TLR signals. We observed that protein levels of both PCBP1 and PCBP2 were unchanged by TLR stimulation (data not shown), suggesting that mRNA destabilization may be caused by the dissociation of PCBP1 and PCBP2 with CRE. Although previous studies have demonstrated that phosphorylation of PCBP1 results in the loss of polyribonucleotide binding ability 21, 22, we did not observe any phosphorylated PCBP1 or PCBP2 after TLR9 stimulation in macrophages (data not shown), suggesting that phosphorylation of PCBP1 and/or PCBP2 did not trigger destabilization of sortilin mRNA in response to TLR signals.

Both PCBPs are iron chaperones that can bind and deliver iron, in addition to their functions as RBPs 23. Interestingly, we observed no relationship between the poly(C)‐ and iron‐binding abilities of either PCBP1 17 or PCBP2 (Fig 4). We have previously shown that Zn^2+^ affected the poly(C)‐binding ability of PCBP1 17; in this study, we obtained similar results with PCBP2 (Fig. 4), suggesting that both PCBP1 and PCBP2 can bind Zn^2+^. We have also demonstrated that cells, which were loaded with Zn^2^+, have decreased sortilin levels 17. Given that TLR signals induce an increase in the cytosolic free Zn^2+^ content 24, 25, we propose a model in which a TLR‐zinc signaling axis triggers dissociation of the PCBP1/2‐mRNA complex by the binding of Zn^2+^ to PCBP1/2, destabilizing sortilin mRNA to attenuate the immune response by preventing cytokine overproduction. We infer that both PCBP1 and PCBP2 may function as sensors of intracellular Zn^2+^ levels to control multiple genes, in addition to *Sort1*, by the binding to their CREs; however, further studies are required to better understand the underlying mechanisms. In summary, we show that the post‐transcriptional regulation of sortilin mRNA by PCBP2 and PCBP1 is important for the post‐translational control of cytokine production.

## Methods

### Mice

C57BL/six mice (female, 6–7 weeks old) were purchased from Sankyo Labo Service Corporation (Tokyo, Japan). All animal experimental protocols were approved by the Animal Research Committee of the Medical Research Institute, Kanazawa Medical University. All animal experiments were carried out in accordance with institutional guidelines.

### Cell culture and reagents, preparation of primary cells from mice

All cells were cultured at 37 °C in a humidified, 5% CO_2_ atmosphere. Media for primary culture were described previously 17. The preparation of primary cells from mice was done as described previously 17. RAW 264.7 cells were grown in Dulbecco's modified Eagle's medium (Wako Chem., Osaka, Japan) supplemented with 10% FCS (Sigma‐Aldrich, St Louis, MO, USA), 100 U·mL^−1^ penicillin, and 100 U·mL^−1^ streptomycin (Wako Chem.).

### Protein depletion by siRNA and immunoblotting

PCBP1 depletion and PCBP2 depletion in RAW264.7 cells were performed by transfection of siRNA (siGENOME SMARTpool siRNA; Dharmacon, Lafayette, CO, USA) using ScreenFect siRNA (Wako Chem.) according to the manufacturer's instructions. A nontargeting sequence siRNA pool (siGENOME Non‐Targeting siRNA Pool #2; Dharmacon Inc) was used as a control. The preparation of whole‐cell lysate and immunoblotting was carried out as previously described 17. The blots were incubated with anti‐PCBP1 (1 : 10 000) 9, anti‐PCBP2 (MBL, Nagoya, Japan; 1 : 1000), and anti‐actin (Sigma‐Aldrich; 1 : 10 000) antibodies. Enhanced chemiluminescent detection was carried out as previously described 17.

### Quantitative real‐time PCR

Total RNA from 5 × 10^5^ cells was isolated with ReliaPrep RNA Cell Miniprep System (Promega, Madison, WI, USA), and cDNA was synthesized using 500 ng of total RNA with ReverTra Ace qPCR RT Master Mix (Toyobo, Ohtsu, Japan), according to the manufacturers' instructions. qRT‐PCR was performed as previously described 17. Cycle threshold values were normalized to the housekeeping gene *Gapdh*. The primer set for the detection of *Pcbp1*, *Sort1*, and *Gapdh* was described previously 17. The primer set for detection of PCBP2 was designed using Primer3Plus (https://primer3plus.com/cgi-bin/dev/primer3plus.cgi): forward, 5′‐TATGCCATTCCACAGCCAGA‐3′; reverse, 5′‐CTGCCCAATAGCCTTTCACC‐3′.

### Electrophoretic mobility shift assay

RNA EMSA with macrophage cell lysate and EMSA with a yeast pep4Δ strain transformed with pYES2 FLAG‐PCBP2 were performed as described previously 17. Antibody supershift experiments were performed by incubation with 1 μg of anti‐PCBP1 or anti‐PCBP2 antibody (MBL) on ice for 5 min after the RNA/protein binding reaction. Normal rabbit IgG (Merck Millipore, Billerica, MA, USA) was used as a negative control. Ferrous ammonium sulfate or zinc sulfate (both 100 μm) was incubated with cell lysate prior to incubation with probes. Samples were separated on 6% polyacrylamide DNA gels (Invitrogen, Waltham, MA, USA) and analyzed by the Odyssey system or phosphorimaging.

### RNA immunoprecipitation and RiboTrap analysis

RNA immunoprecipitation was carried out as described previously 17. Briefly, 60 µL of Magnetic Beads Protein A/G (Merck Millipore) bound to 15 µg of anti‐PCBP2 (MBL) or rabbit IgG (Merck Millipore) was incubated with lysate at 4 °C for 18 h. RNA precipitated by antibodies was purified using an miRNeasy Mini Kit (QIAGEN, Hilden, Germany), followed by cDNA synthesis with iScript Advanced cDNA Synthesis Kit (Bio‐Rad Laboratories, Richmond, CA, USA). Real‐time PCR was carried out using a GoTaq Master Mix (Promega).

The cytosolic fraction of macrophages for RiboTrap analysis was prepared from 5 × 10^7^ macrophages using a RiboCluster Profiler RiboTrap kit (MBL), according to the manufacturer's instructions. For *in vitro* transcription with T7 RNA polymerase, the 3′ UTR of sortilin mRNA was amplified from a plasmid containing full‐length sortilin cDNA (DNAFORM, Yokohama, Japan), and the resulting amplicon was cloned into a pGEM‐4Z vector (Promega) using the In‐Fusion HD Cloning Kit (Clontech, Palo Alto, CA, USA). The BrUTP‐labeled 3′ UTR of sortilin mRNA was prepared using an RNA Riboprobe System‐T7 kit (Promega). Magnetic Beads Protein A/G (Merck Millipore) bound to anti‐BrdU (MBL) or rabbit IgG (Merck Millipore) were incubated with the mixture of cytosol and BrUTP‐labeled 3′ UTR at 4 °C for 3 h. The BrUTP‐labeled RNA–protein complex bound to magnetic beads was eluted with PBS containing BrdU, followed by immunoblotting.

### Statistical analysis

Statistical analysis was performed by ANOVA using graphpad prism software (GraphPad Software, La Jolla, CA, USA). One‐way and two‐way ANOVA were used for experiments with one and two variables, respectively. A *P* value of < 0.05 was considered statistically significant.

## Conflict of interests

The authors declare no conflict of interest.

## Author contributions

TY‐W conceived the project. TY‐W, CCP, and NO supervised the project. TY‐W performed the experiments and analyzed the results. TY‐W, CCP, and NO wrote and edited the manuscript.
